# Important CT findings for prediction of severe appendicitis: involvement of retroperitoneal space

**DOI:** 10.1186/1749-7922-9-62

**Published:** 2014-12-17

**Authors:** Kumiko Kitaoka, Kazuhiro Saito, Koichi Tokuuye

**Affiliations:** Department of Radiology Tokyo Medical University Hospital, 6-7-1 Nishishinjuku, Shinjuku-ku, Tokyo, 160-0023 Japan

**Keywords:** Appendicitis, Complicated, Retroperitoneal space, Extraperitoneal space, CT

## Abstract

**Background:**

Determination of the severity of appendicitis and differentiation between complicated and uncomplicated appendicitis are clinically important. Severe appendicitis frequently affects extraperitoneal spaces. We have investigated CT findings of retroperitoneal space (RPS) in patients with appendicitis to create a model for identification of complicated appendicitis.

**Method:**

CT images of 223 patients with pathologically proven appendicitis were reviewed. The total number of the segments in RPS where inflammatory changes were located (RPS count) was obtained as well as appendiceal diameter, appendicolithiasis, WBC count, and CRP level. Data were analyzed to identify factors indicating complicated appendicitis. Univariate analysis was conducted to identify statistically significant variables. A multivariable logistic regression analysis was performed in order to find independent predictors of complicated appendicitis.

**Results:**

Patients with complicated appendicitis were more likely to have higher RPS count (P < 0.001), appendicolithiasis (P = 0.002), higher CRP level (P < 0.001), and greater appendix diameter (P < 0.001) than patients with uncomplicated appendicitis. Statistical analysis showed RPS count was the most helpful predictor of complicated appendicitis.

**Conclusion:**

Radiologists and surgeons should be aware of the importance of CT findings in RPS when treating patients with appendicitis. Complicated appendicitis can be predicted by RPS count, diameter of the appendix, appendicolithiasis, and CRP level.

## Introduction

Appendicitis, a very common surgical condition, has been traditionally considered as a surgical emergency and a clinical challenge [1]. With the advent of computed tomography (CT), the diagnostic accuracy of appendicitis has dramatically improved. So far, CT findings, such as enlargement of the appendix, appendicolithiasis, and phlegmon, have been reported to successfully identify cases of acute appendicitis and these findings are relatively easy to identify [2–4]. Other researchers have reported that laboratory markers are useful in making diagnosis [5]. Recently, one systematic review and meta-analysis reported imperfect accuracy of procalcitonin, C-reactive protein (CRP) and white blood cell count (WBC) in uncomplicated or complicated appendicitis [6].

In patients with complicated appendicitis, a significant increase in mortality rate has been reported [7, 8]. Accordingly, accurate determination of the severity of appendicitis, and differentiation between complicated and uncomplicated appendicitis is clinically warranted. CT could help surgeons recognize the location of appendix, confirm the diagnosis and other intra-abdominal conditions requiring other procedures, such as perforation, abscess, and peritonitis [9]. Some previous studies have investigated the implication of various CT findings in the context of severity of appendicitis [10–17]. Clinical application of such findings, however, may not be easy, because they can be subtle, and subjective.

The appendix is usually located in the anterolateral portion of pelvic cavity, and inflammation of the appendix can trigger inflammatory process in pelvic extraperitoneal space (PEPS). Recent studies have discovered more precise anatomical relationship between retroperitoneal space (RPS) and PEPS [18–20].

## Methods

Approval was obtained by the institutional review board of our university before initiation of this study. All consecutive patients who underwent an appendectomy for suspected acute appendicitis at our university hospital between January 1, 2007 and December 31, 2012 were eligible. All eligible patients were chosen by searching a pathology database with an appropriate code for appendix or appendectomy. The eligibility criteria were a histology report showing acute appendicitis (n = 231). Patients were excluded when pathologists made a diagnosis of chronic appendicitis (n = 7), or when there was no preoperative, non-focused, abdominopelvic CT examination available in our picture archiving and communication system (PACS) at the time of the study that had been obtained before a surgery (n = 1). Written informed consent was obtained from the patient on admission for the publication of clinical report and any accompanying images performed during their medical treatment.

We reviewed the patients’ medical, surgical, and pathology records. All multidetector non-contrast or contrast CT images were obtained to visualize structures in ileocecal area. All patients were scanned using a 16 or 64 multi-detector helical scanner, (Bright Speed Elite; Right Speed VCT General Electric Health Care). The abdomen was scanned helically (pitch of 1.375:1, rotation time 0.4 sec, 120kVp, auto mAs) with a 5-mm collimation.

The images were retrospectively reviewed at a PACS workstation and interpreted by consensus of two radiologists (K.S. 23 years of experience, specialized in abdominal radiology; K.K. 3 years of experience) who were blinded to the patients’ surgical or pathological results.

**Figure 1:**
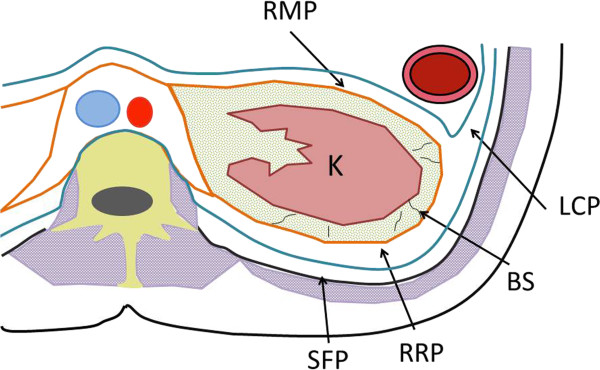
**Transverse diagram showing segments in RPS.** Retromesenteric plane (RMP), lateral conal plane (LCP), retrorenal plane (RRP), subfascial plane (SFP), combined fascial plane (CFP), and bridging septa (BS) constitute retroperitoneal space (RPS) around the kidney (K). RMP is an interfascial space bounded by the anterior pararenal space and the perirenal space. RRP is located between the perirenal space and the posterior pararenal space. RMP and RRP extend caudally to form CFP below the kidney. RMP and RRP continue anterolaterally to LCP. BP is a lamellar structure having connection with the anterior and posterior renal fasciae. SFP is a potential space between the pararenal space and the transversalis fascia.

**Figure 2:**
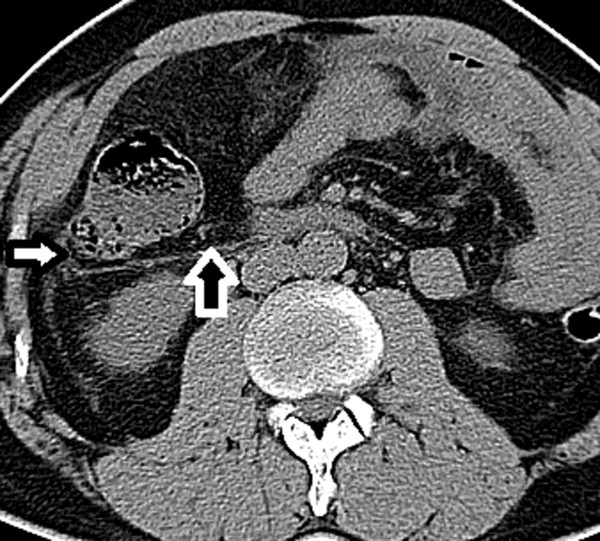
**Transverse CT image of 46-year-old man with complicated appendicitis.** Retrorenal plane, retromesenteric plane (black arrow), lateral conal plane (white arrow), subfascial plane, and bridging septa are involved.

**Figure 3:**
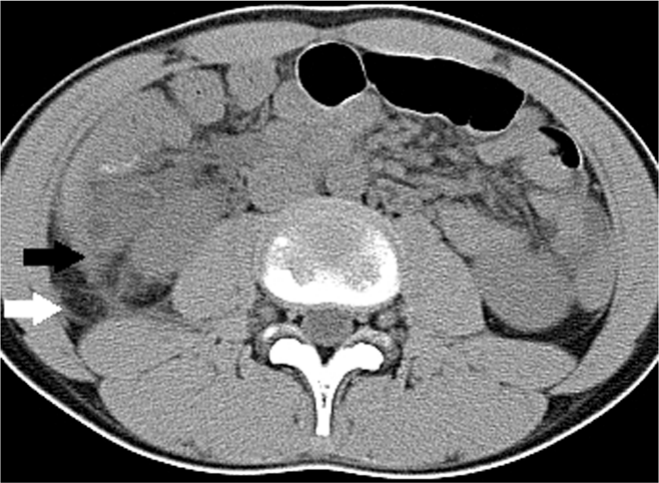
**Transverse CT image of 13-year-old boy with complicated appendicitis.** The picture shows enlarged appendix with fluid collection in retrorenal plane, subfascial plane (white arrow), lateral conal plane (black arrow), and bridging septa.

We selected six sections within RPS without discriminating the right from the left. These sections included (a) lateral conal plane (LCP), (b) retromesenteric plane (RMP), (c) retrorenal plane (RRP), (d) bridging septa (BS), (e) subfascial plane (SFP), and (f) combined fascial plane (CFP) [21] (Figures 1, 2 and 3). These segments were evaluated for signs of inflammation, such as thickening of the fascial plane (Figures 2 and 3). We regarded recognizable thickening of fascial planes of any degree as pathological as well as fluid accumulation. RPS segments where signs of inflammation could be located and the total number of involved RPS segments (RPS count) were recorded for each patient. In addition, maximal diameter of the appendix on an axial image was measured, and presence or absence of appendicolithiasis was determined. Patients were divided into two groups a complicated appendicitis group and an uncomplicated appendicitis group. The complicated appendicitis group consisted of patients whose surgical reports either described the presence of a perforated appendix, abscess formation, or purulent peritoneal fluid, or whose histology reports indicated a perforated appendix, abscess formation, peritonitis, or gangrenous appendicitis.

We analyzed data using the Statistical Package for the Social Sciences (SPSS) ver. 16.0 (SPSS Inc., Chicago, IL). Univariate analysis was performed by comparing characteristics between complicated and uncomplicated appendicitis. Analysis was also repeated for two subgroups, the younger patients (age <16 years, n = 68) and the older patients (age ≧16 years, n = 155).

A multivariable logistic regression analysis was performed in order to find factors for indicating complicated appendicitis.

## Results

A total number of 223 patients with pathologically proven appendicitis, 78 female, 145 male, were included. The mean age of the patients was 31.4 years old (4–94 year old). 123 patients had complicated appendicitis (55.2%).

**Figure 4:**
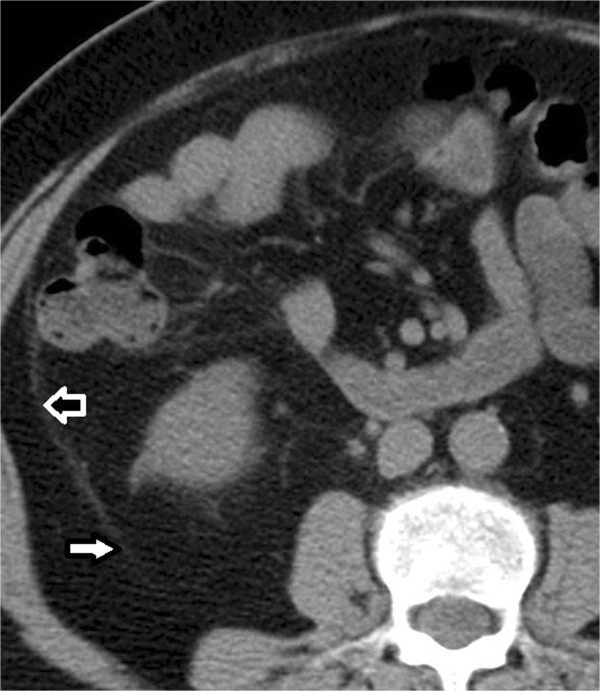
**Transverse CT image of 59-year-old man with complicated appendicitis.** Retromesenteric plane, retrorenal plane (white arrows), and lateral conal plane (black arrow) were involved.

**Figure 5:**
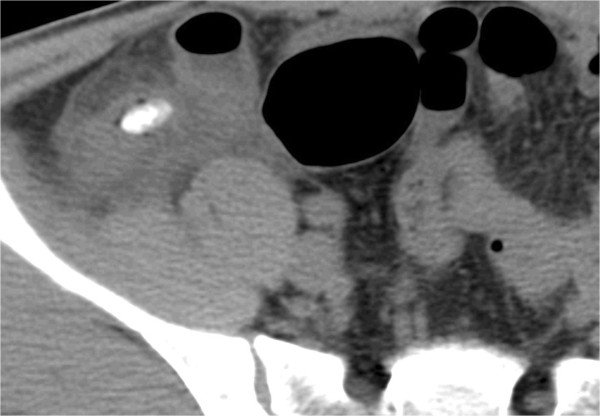
**Transverse CT image of 27-year-old man with complicated appendicitis.** Appendicolithiasis was observed with involvement of combined fascial plane.

**Figure 6:**
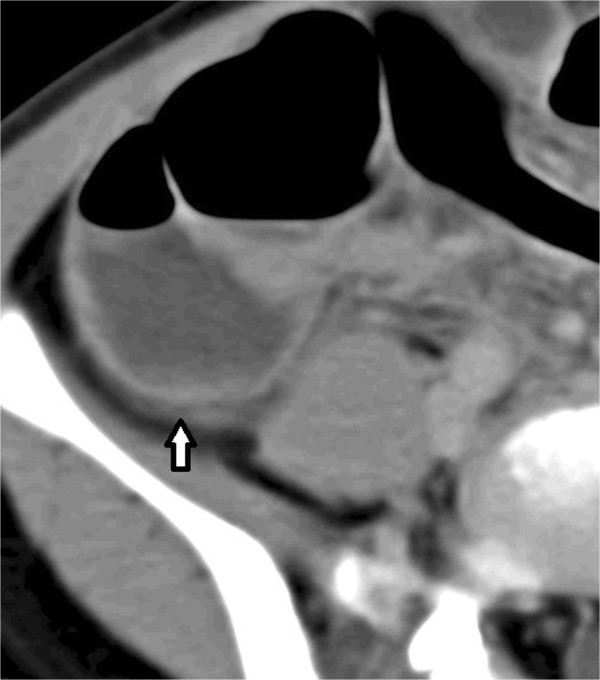
**Transverse CT image of 6-year-old patient with complicated appendicitis and ileus.** Identification of RPS and other extraperitoneal spaces was difficult due to the patient’s younger age and ileus. Combined fascial plane (white arrow) involvement was observed.

**Figure 7:**
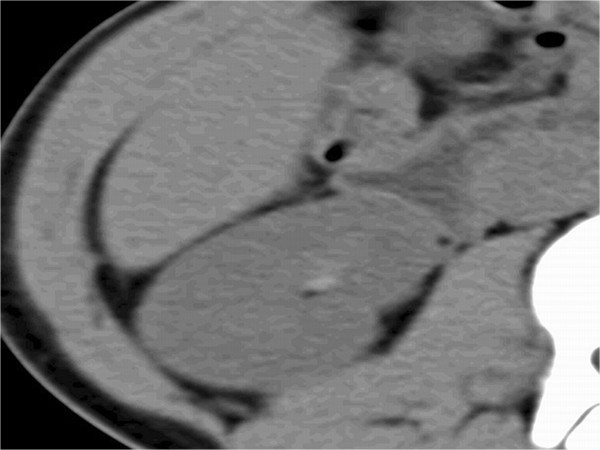
**Transverse CT image of 12-year-old patient with phlegmonous appendicitis.** No change can be observed in RPS.

Observed distribution of changes in each segment of RPS for the complicated/uncomplicated appendicitis group was summarized (Table 1). Significantly more RPS segments were involved in the complicated appendicitis group compared with the uncomplicated appendicitis group (Figures 2, 3, 4 and 5). Involvement of RPS segments was more identifiable in the older patient (age ≧16 years) group than in the younger patient group (age <16 years) because segments of RPS are sometimes difficult to be observed (Figures 6 and 7).

**Table 1:** **Distribution of observed radiological changes in RPS segments**

		LCP	RRP	RMP	BS	SFP	CFP
Complicated appendicitis (true positive)	≧16y	49	38	33	12	12	82
	<16y	4	5	3	1	5	17
Uncomplicated appendicitis (false positive)	≧16y	7	7	5	2	1	46
	<16y	6	3	3	0	0	15
PPV	≧16y	0.88	0.84	0.87	0.86	0.92	0.64
	<16y	0.40	0.63	0.50	1.0	1.0	0.53
NPV	≧16y	0.53	0.47	0.46	0.40	0.41	0.48
	<16y	0.60	0.63	0.61	0.61	0.65	0.72

Significantly more segments were involved (i.e. radiographical changes were observed) in complicated appendicitis group. CFP, which is closest to the pelvic extraperitoneal space, was predominantly involved. Positive predictive value (PPV) of complicated appendicitis was highest for SFP in older patient group while PPV was highest for SFP and BS in younger patient group.

Positive predictive value (PPV) was relatively high (0.64-0.92) for segments of RPS among older patients. The most frequently involved segment was CFP, which had Negative predictive value (NPV) of 0.72 among younger patients. The least frequently involved segment, SFP had PPV of 0.92 among older patients (Table 1).

Univariate analysis revealed that patients with complicated appendicitis were more likely to have higher RPS count (P < 0.001), higher CRP level (P < 0.001), greater appendix diameter (P < 0.001), and appendicolitiasis (P = 0.002) (Table 2). No statistically significant differences were noted between both groups with respect to gender and WBC count.

**Table 2:** **Results of univariate analysis of variables between groups**

	Complicated (n = 123)	Uncomplicated (n = 100)	P value
Appendix diameter*^†^ (mm)	13.4 (10.4-16.0)	10.6 (8.6-13.1)	<0.001
RPS count*^†^	2 (1-3)	1(0-1)	<0.001
WBC count^†^ (×10^9^ cells/L)	13.7 (11.3-17.0)	13.7 (11.1-16.5)	>0.05
CRP level*^†^ (nmol/L)	45.7 (11.4-131.4)	12.4 (4.8-36.2)	<0.001
Age*^†^(years old)	34(18-51)	23 (11-34.8)	<0.001
Appendicolithiasis*^‡^			0.002
Gender^‡^			>0.05

*P value <0.05 was considered significant.

†Medians are shown with 25th percentile and 75th percentile between brackets.

‡Data were analyzed using Fishers’ exact test.

In the older patient group, patients with complicated appendicitis were more likely to have higher RPS count (P < 0.001), higher CRP level (P < 0.001), greater appendix diameter (P = 0.002), appendicolitiasis (P = 0.04), and advanced age (P = 0.01). In the younger patient group, patients with complicated appendicitis were more likely to be female (P = 0.02), more likely to have appendicolitiasis (P = 0.01), greater appendix diameter (P = 0.02), and higher CRP level (P = 0.03).

Variables of age, RPS count, diameter of appendix, appendicolithiasis, and CRP level were entered into the initial logistic regression model.

The final model included RPS count, diameter of appendix, appendicolithiasis, and CRP level, and was characterized by a Nagelkerke R-square value of 0.31 and a Hosmer and Lemeshow level of fit with a χ2 value of 7.9 and a P value of 0.45.

**Figure 8:**
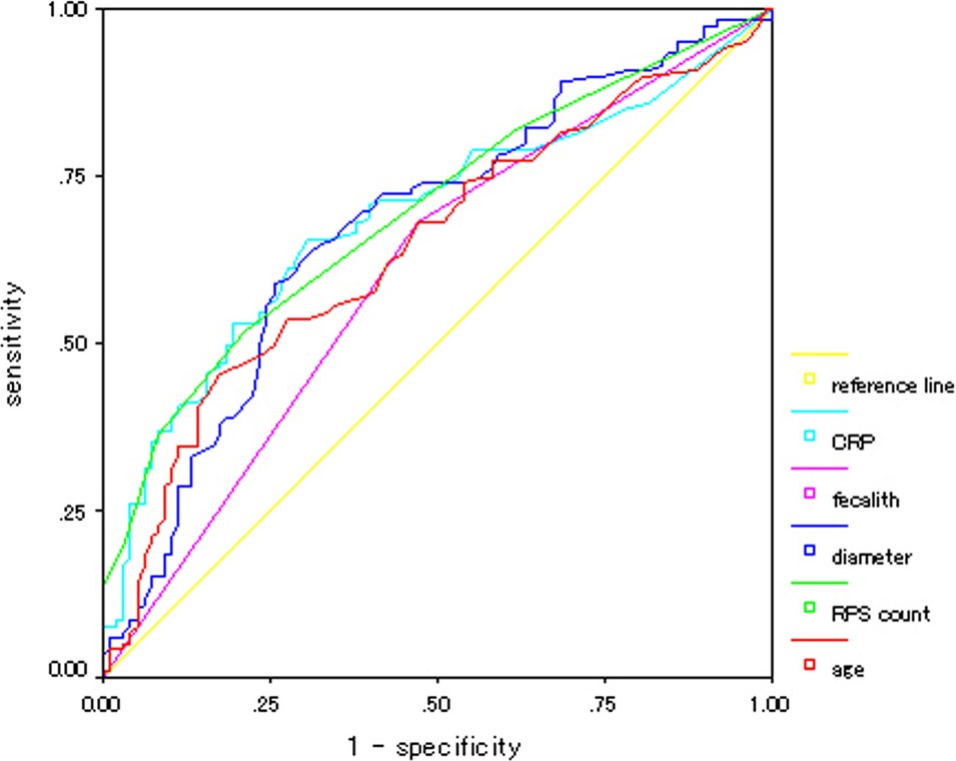
**Receiver characteristic curve derived from sensitivities and specificities of various clinical variables.** RPS count is plotted in light green line with the following variables: CRP level (light blue), appendicolithiasis (pink), appendiceal diameter (dark blue), and age (red). Yellow line is a reference line. RPS count had the largest area under the curve of 0.70.

The regression coefficients for the final regression models are shown in Table 3. Obtained ROC curve showed the largest area under the curve (AUROC) for RPS (AUROC = 0.70), followed by CRP level (AUROC = 0.69) (Figure 8). The optimal cutoff value of RPS count for complicated appendicitis was calculated around 1 to 2 (sensitivity = 0.521, specificity 0.786 at RPS = 1.5).

**Table 3:** **Logistic regression model for probability of complicated appendicitis**

	P value	Regression coefficient	Odds ratio
RPS count	0.004	0.419	1.52
Appendix diameter	0.06	0.088	1.09
Appendicolithiasis	0.09	0.863	0.42
CRP level	0.08	0.076	1.709

Analysis was repeated for the older patient group and RPS count was found to be a better indicator of complicated appendicitis than other variables. The final model included only RPS count, appendicolithiasis, and CRP level. AUROC was largest for RPS (AUROC = 0.71).

## Discussion

This study showed a complicated appendicitis can be predicted by a combination of clinical variables, the number of involved segments of RPS, the maximal diameter of the appendix on CT examination, existence of appendicolithiasis, and the CRP levels. Based on our data, CT analysis has strong predictive power for the severity of appendicitis [21]. This finding is similar to the pancreatitis severity scoring system [22], which determines the severity of pancreatitis based on the extent of inflammation on CT. The appendix, being located in the proximity of PEPS, can cause inflammation-related CT changes in RPS as well by way of CFP.

Compared with laboratory markers, CT findings of appendicitis have been found helpful because of their specificity. Noncontrast CT scan in adults had reasonably high sensitivity and specificity for clinical decision making. Previous radiologists who studied severity of appendicitis limited the scope of observation to findings observed in the vicinity of the appendix, such as involvement of periappendiceal fat and appendicolithiasis [9–17]. Although these results are important, these findings are not always reproducible. For example, contrast medium is contraindicated in some patients while many of these findings are sometimes difficult to be identified without contrast enhancement. Furthermore, not every clinician is familiarized with subtle CT changes. Contrary to these limitations, the key elements of CT findings that we presented in this article can be interpreted more objectively and in a semiquantitative manner in almost every patient once clinicians know where to look at.

The present study included 123 cases of complicated appendicitis in 223 cases of pathologically proven appendicitis (55.2%). The ratio of complicated appendicitis is relatively high compared with other previous reports (20-40%) [9, 16, 17]. There was no appendicitis-associated death observed among the study subjects.

Potential limitations of this study include difficulty of identifying anatomical structures in pediatric patients. We acknowledge that this method may be more useful in patients who are at the age of 16 or higher. CT exposes patients to a risk of ionizing radiation. Therefore, CT is less indicated in the young and female patients due to its high level of irradiation and may be more helpful in the elderly age group, but there is still a 10-20% negative appendectomy rate despite the use of CT [23–25]. However, most importantly we can utilize this diagnostic method without specialized training or it can be employed irrespective of use of contrast medium once its practical utility is properly understood.

## Conclusion

In conclusions, radiographical findings of RPS segments are important for the diagnosis of appendicitis and the prediction of severity of acute appendicitis because numerosity of the involved RPS segments (for example ≧2) is indicative of severe appendicitis. Complicated appendicitis can be predicted by RPS count, diameter of the appendix, appendicolithiasis, and CRP level. The possibility of false positivity remains, but RPS count would be more useful than other CT findings because administration of contrast medium is not required and it can be utilized in almost every clinical setting. Radiologists and clinicians should continue appropriate measures to minimize irradiation for susceptible patients.
